# The genus *Arhaconotus* Belokobylskij (Hymenoptera, Braconidae, Doryctinae) from China, with description of a new species

**DOI:** 10.3897/zookeys.61.488

**Published:** 2010-10-11

**Authors:** Pu Tang, Jun-Hua He, Xue-Xin Chen

**Affiliations:** State Key Laboratory of Rice Biology, Institute of Insect Science, College of Agriculture and Biotechnology, Zhejiang University, Hangzhou 310029, China

**Keywords:** Hymenoptera, Braconidae, Doryctinae, Araconotus, new species, new record, Oriental region, China

## Abstract

The genus Arhaconotus Belokobylskij (Hymenoptera: Braconidae, Doryctinae) is recorded for the first time from China. A new species of this genus, Arhaconotus hainanensis Tang & Chen, **sp. n.**, is described and illustrated. A key to the species of this genus is updated to include the new species.

## Introduction

The genus Arhaconotus Belokobylskij (Hymenoptera, Braconidae, Doryctinae) was described in 2000 by S.A. Belokobylskij (Belokobylskij 2000). To date three species of the genus have been described, of which one occurs in the Australasian region and two in the Oriental region (Belokobylskij 2000, 2001). During our study of Chinese Braconidae, we discovered a new species of this genus. It represents the first record of this genus from China. In the present paper, the new species, Arhaconotus hainanensis Tang & Chen, sp. n., is described and illustrated and an updated key to the genus Arhaconotus is added.

## Material and methods

The terminology and measurements used follow van Achterberg (1979, 1988). Additional sources for the description of sculpture and setation are Belokobylskij (2001). All descriptions and measurements were made under a Leica MZ 12.5 microscope, and all figures were made by a digital camera (Q-Imaging, Micropublisher, 3.3 RTV) attached to a stereomicroscope (Leica MZ APO, Germany) and Auto-Montage Pro version 5.0 software. Type specimens and other materials are deposited in the Parasitic Hymenoptera Collection of the Zhejiang University, Hangzhou, China (ZJUH).

## Taxonomy

### Arhaconotus hainanensis

Tang & Chen sp. n.

urn:lsid:zoobank.org:act:C2DEBB99-C921-4CE8-AE2E-E1DEE8ABCB50

Figs 1–8 

#### Material examined.

Holotype: ♀, China, Hainan Prov., Bawangling, 9–10.VI.2007, Liu Jingxian, No. 200703484 (ZJUH). Paratypes: China, Hainan Prov.: 1♀, Jianfengling, 5–7.VI.2007, Weng Liqiong, No. 200806631; 1♀, Jianfengling Tianchi, 22–23.X.2007, Liu Jingxian, No. 200710486 (ZJUH).

#### Description.

##### Female.

###### Body length

3.2 mm; fore wing length 2.9 mm.

###### Head.

Width 1.4 times its median length. Antennae slender, almost filiform, 32-segmented. Scapus 1.6 times as long as maximum width. First flagellar segment 6 times as long as its apical width, almost equal to second segment. Penultimate segment 4.8 times as long as wide, 0.7 times as long as first segment, 0.9 times as long as apical segment. Eye 2.6 times as long as temple in dorsal view. Ocelli medium-sized, in triangle with base almost equal to its sides. POL: OD: OOL= 3: 3: 6. Temple finely granulate ventrally. Vertex and frons densely granulate. Vertex with very sparse, short setosity. Eye glabrous, 1.2 times as high as broad. Face finely granulate-coriaceous, its width 1.2 times height of eye, 1.4 times height of face and clypeus combined. Malar space 0.3 times height of eye, 0.7 times as long as basal width of mandible. Malar suture absent. Occipital carina complete dorsally, not fused with hypostomal carina ventrally.

###### Mesosoma.

Length 2.3 as long as its height. Pronotal carina fine, distinctly separated from posterior margin of pronotum; distances from carina to posterior and anterior margins of pronotum subequal. Mesoscutum entirely densely and evenly pubescent, densely granulate, highly and roundly raised above pronotum; its median lobe without median depression. Notauli deep, complete, crenulate. Scutellum densely granulate. Prescutellar depression deep, 0.5 times as long as scutellum. Mesopleuron densely coriaceous and its upper third longitudinally striate. Precoxal sulcus deep, coriaceous, weakly curved, running along anterior 2/3 of mesopleuron, connected with prepectal carina. Prepectal carina distinct, wide ventrally, without widened lobes opposite to fore coxa. Propodeum with median carina in basal third, and without marginate areola; basolateral areas distinctly marginate, coriaceous; rest part of propodeum rugulose laterally and in posterior half.

###### Wings.

Fore wing 3.3 times as long as maximum width. Vein r arising slightly before middle of pterostigma. 3-RS forming very obtuse angle with r. 3-RS: r: SR1= 24: 9: 40. Second submarginal cell large, 3 times as long as maximum width, 1.4 times as long as first subdiscal cell, almost equal to first discal cell. 1-SR+R weakly S-curved. m-cu postfurcal. 1-CU1 almost equal to cu-a. Cu1a interstitial. Hind wing, M+CU 0.6 times as long as 1-M. m-cu weakly curved, antefurcal and pigmented.

###### Legs.

Hind coxa granulate. Hind femur coriaceous, 3.3 times as long as wide, with weak dorsal protuberance. Hind tibia with rather long, dense setae dorsally. Hind tarsus almost as long as hind tibia. Hind basitarsus 0.8 times as long as second-fifth segments combined; second tarsal segment 0.4 times as long as basitarsus, 1.3 times as long as fifth segment (excluding pretarsus).

###### Metasoma.

Almost as long as mesosoma and head combined, with 6 visible tergites. First tergite entirely, distinctly longitudinally striate, its apical width 1.6 times its minimum width; its length 1.2 times as long as its maximum width. At most part of second tergite distinctly longitudinally striate, with a distinctly separated, smooth basal area and a rather wide smooth apical area, median length of second tergite 0.7 times as long as its basal width. Second suture deep and wide. Second-fifth tergites densely striate-punctulate in entire lateral parts. Third-fifth tergites in basal halves (their apical halves smooth) distinctly longitudinally striate. Sixth tergite rather large, densely punctulate on basal half, semicircularly striate on apical half, regularly rounded on apical margin with a shallow median emargination. Ovipositor sheath 1.2 times as long as metasoma and 0.6 times as long as fore wing.

###### Colour.

Head reddish yellow. Mesonotum and apex of metasoma reddish brown; rest part of mesosoma and metasoma black. Basal quarter of antenna reddish brown, remainder dark reddish brown to black. Palpi pale yellow. Legs entirely yellow, sometimes hind coxa infuscate. Ovipositor sheath dark brown, paler basally. Wings faintly infuscate. Pterostigma brown, yellow in basal third and in apical 1/4.

**Figures 1–10.**
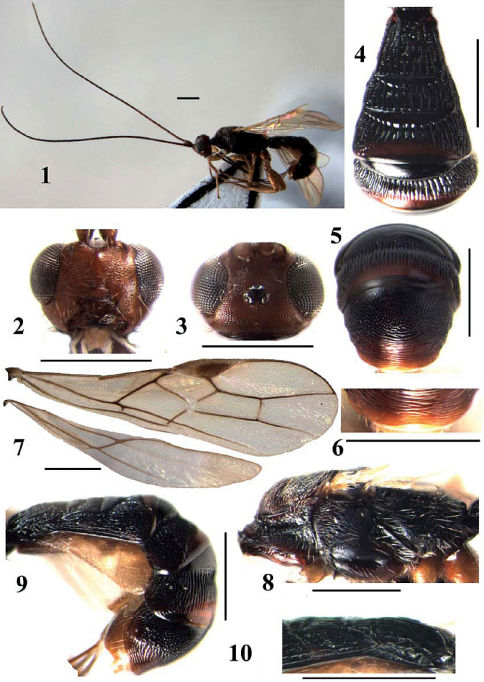
Arhaconotus hainanensis sp. n. **1** habitus, lateral aspect **2** head, frontal aspect **3** head, dorsal aspect **4** first-fourth abdominal tergites, dorsal aspect **5** fifth-sixth abdominal tergites, dorsal aspect **6** posterior margin of the sixth tergite, dorsal aspect **7** fore and hind wings **8** mesosoma, lateral aspect **9** metasoma, lateral aspect **10** first and second tergites,lateral aspect.scale bar: 5mm

##### Male.

Unknown.

##### Variation.

Body length 2.8–2.9 mm; fore wing length 2.6–2.7 mm. Fore wing 3.9 times as long as maximum width, 3-RS: r: SR1=21: 6: 39. Second submarginal cell 3 times as long as maximum width. Median length of second tergite 0.8 as long as its basal width.

#### Diagnosis.

This new species is similar to Arhaconotus vietnamicus Belokobylskij, but differs in antenna longer; sixth tergite densely punctulate on basal half and semicircularly striate on apical half, its apical margin with a shallow median emargination; second to fifth tergites densely striate-punctulate in entire lateral parts, and metasoma black.

#### Distribution.

China (Hainan).

#### Etymology.

From the Hainan province, type locality of the species.

#### Key to species of genus Arhaconotus Belokobylskij

.

**Table:** 

1.	Vertex and most part of mesosoma smooth; sixth tergite without medio-posterior emargination; propodeum with marginate areola	Arhaconotus papuanus Belokobylskij
–	Vertex granulate or coriaceous and most part of mesosoma granulate; sixth tergite with medio-posterior emargination; propodeum without marginate areola	2
2.	Ovipositor sheath longer, 0.85 times as long as fore wing; lateral lobes of mesoscutum glabrous at large part; hind femur 3.8 times as long as wide	Arhaconotus ishigakiensis Belokobylskij
–	Ovipositor sheath shorter, 0.6–0.65 times as long as fore wing; lateral lobes of mesoscutum entirely setose; hind femur 3–3.3 times as long as wide	3
3.	Sixth tergite densely punctulate on basal half and semicircularly striate on apical half, its apical margin with a shallow median emargination; second-fifth tergites densely striate-punctulate in entire lateral parts; metasoma black; antenna 32-segmented	Arhaconotus hainanensis sp. n.
–	Sixth tergite entirely semicircularly striate, its apical margin with a distinct median emargination; second-fifth tergites densely striate in entire lateral parts; metasoma reddish brown; antenna 27-segmented	Arhaconotus vietnamicus Belokobylskij

## Supplementary Material

XML Treatment for Arhaconotus hainanensis
